# Exploration of methylation-driven genes for monitoring and prognosis of patients with lung adenocarcinoma

**DOI:** 10.1186/s12935-018-0691-z

**Published:** 2018-11-26

**Authors:** Chundi Gao, Jing Zhuang, Huayao Li, Cun Liu, Chao Zhou, Lijuan Liu, Changgang Sun

**Affiliations:** 1 College of First Clinical Medicine, Shandong University of Traditional Chinese Medicine, Jinan, 250014 Shandong People’s Republic of China; 2 Department of Oncology, Affiliated Hospital of Weifang Medical University, Weifang, 261031 Shandong People’s Republic of China; 3 Departmen of Oncology, Weifang Traditional Chinese Hospital, Weifang, 261041 Shandong People’s Republic of China; 4 College of Traditional Chinese Medicine, Shandong University of Traditional Chinese Medicine, Jinan, 250014 Shandong People’s Republic of China

**Keywords:** Lung adenocarcinoma, DNA methylation-driven genes, Biomarkers, Cox proportional hazards regression, Survival analysis

## Abstract

**Background:**

As one of the most common malignant tumors in humans, lung cancer has experienced a gradual increase in morbidity and mortality. This study examined prognosis-related methylation-driven genes specific to lung adenocarcinoma (LUAD) to provide a basis for prognosis prediction and personalized targeted therapy for LUAD patients.

**Methods:**

The methylation and survival time data from LUAD patients in the TCGA database were downloaded. The MethylMix algorithm was used to identify the differential methylation status of LUAD and adjacent tissues based on the β-mixture model to obtain disease-related methylation-driven genes. A COX regression model was then used to screen for LUAD prognosis-related methylation-driven genes, and a linear risk model based on five methylation-driven gene expression profiles was constructed. A methylation and gene expression combined survival analysis was performed to further explore the prognostic value of 5 genes independently.

**Results:**

There were 118 differentially expressed methylation-driven genes in the LUAD tissues and adjacent tissues. Five of the genes, CCDC181, PLAU, S1PR1, ELF3, and KLHDC9, were used to construct a prognostic risk model. Overall, the survival time was significantly lower in the high-risk group compared with that in the low-risk group (P < 0.05). In addition, the methylation and gene expression combined survival analysis found that the combined expression levels of the genes CCDC181, PLAU, and S1PR1 as well as KLHDC9 alone can be used as independent prognostic markers or drug targets.

**Conclusion:**

Our findings provide an important bioinformatic basis and relevant theoretical basis for guiding subsequent LUAD early diagnosis and prognosis assessments.

**Electronic supplementary material:**

The online version of this article (10.1186/s12935-018-0691-z) contains supplementary material, which is available to authorized users.

## Background

Lung cancer is one of the most malignant tumors in the world, with high morbidity and mortality [1]. Epidemiological statistics show that the incidence of lung adenocarcinoma (LUAD) in lung cancer is increasing, and it is more common among women and non-smokers [2, 3]. Due to the existence of tumor heterogeneity factors and the different pathological and molecular types of patients, the personalized treatment of LUAD faces large challenges [4]. In recent years, with the advancement of molecular biology, several LUAD driving genes such as EGFR, KARS, and TP53 have been discovered one after the other [5], and some targeted therapeutic drugs have been developed, which has greatly improved the treatment of some patients with LUAD [6, 7]. However, not all patients have benefited from these drugs, such as those who cannot tolerate the therapeutic side effects of targeted drugs or those who are resistant to drugs in the short term. Therefore, identification of new LUAD-associated driver genes by bioinformatics analysis and construction of risk model are necessary for the prognosis evaluation and post-treatment of patients.

Molecular mechanism research based on bioinformatic analysis is an important method for cancer research. Not only can this research explore the molecular pathogenesis of tumors but also it can identify biomarkers for early diagnosis, treatment and prognosis of tumors [8]. As one of the core elements in epigenetic modification, DNA methylation is an important signaling tool that regulates genomic function and is one of the important features mediating carcinogenesis [9]. DNA methylation can participate in many cellular processes such as cell differentiation, genome stability, and gene imprinting [10, 11]. Biological processes, specifically, changes in DNA methylation, can provide an important basis for early diagnosis and prognosis of cancer and a new ideas for further clinical applications.

In recent years, sufficient research evidence has shown that the occurrence and development of lung cancer is a multi-factor, multi-stage and multi-gene change process in which the inactivation of tumor suppressor gene methylation is one of its important mechanisms [12–15]; consequently, in-depth research on the lung cancer-related methylation mechanism has become a focus of great concern. A large number of studies have shown that the methylation of certain genes can affect their expression, and this phenomenon is closely related to the diagnosis and prognosis of lung cancer [16]. For example, Feng et al. showed that RAR-β methylation levels were abnormally expressed in non-small cell lung cancer (NSCLC) patients, while those with positive APC methylation status in tumor tissues survived longer than those with negative APC methylation, indicating that the methylation of RAR-β or APC is a promising diagnostic or prognostic marker of NSCLC [17]. In addition, Zhang et al. found that the methylation level of PAX6 in non-small cell lung cancer tissues was higher than that in normal tissues and that the methylation status of PAX6 was associated with a poor overall survival rate in cancer tissues. It has been suggested that methylated PAX6 may be a useful biomarker for the prognosis assessment of NSCLC [18].

The Cancer Genome Atlas (TCGA) database [19] provides researchers around the world with open data on cancer genetic and epigenetic profiles, enabling researchers to efficiently capture and analyze relevant data and genomic changes. MethylMix, an algorithm implemented with R, identifies methylation status based on a β-mixed model to identify disease-specific hypomethylated and hypermethylated genes to obtain disease-related methylation-driven genes [20]. At present, related studies on methylation-driven genes have been reported [21]; in this study, the methylation and mRNA expression data of LUAD patients were extracted from the TCGA database, and the methylation-driven genes related to LUAD were obtained by use of the MethylMix algorithm. The Cox–Kaplan–Meier-survival method was used to construct a survival model, assess the methylation-driven genes related to LUAD prognosis, explore the correlation between DNA aberrant methylation and LUAD genome level, and provide a scientific basis for personalized medicine.

## Materials and methods

### Data processing and analysis

We downloaded methylation and mRNA expression data from LUAD patients from the TCGA database. Among them were methylation data from 507 samples, including 32 normal samples and 475 cancer samples, as well as mRNA expression data from 594 samples, including 59 normal samples and 535 LUAD samples. First, based on the LIMMA package, the downloaded data were normalized and analyzed for differences to obtain aberrant methylated genes and differentially expressed genes. Then, based on the MethylMix algorithm implemented by R, we calculated the correlation between gene methylation level and gene expression. Next, we determined genes that were significantly related and identified the disease-specific hypomethylation and hypermethylation genes by constructing the β-mixed model. Finally, screening for methylation-driven genes was done. In addition, we screened 244 samples with stage I LUAD,which both have expression and clinical information for further testing. The data provided by TCGA is public and did not require the approval of a local ethics committee.

### Functional and pathway enrichment analysis of methylation-driven genes

The Database for Annotation, Visualization and Integrated Discovery (DAVID) v6.8 (http://david.abcc.ncifcrf.gov/) serves as an open source platform for determining associations between target molecules [22]. ConsensusPathDB (http://cpdb.molgen.mpg.de/) integrates interaction networks in Homo sapiens, including binary and complex signaling, gene regulatory and drug-target interactions, as well as biochemical pathways [23, 24]. To gain insight into the biological functions of these methylation-driven genes, the genes were subjected to functional and pathway enrichment analyses based on DAVID and Consensus PathDB online software, and P < 0.05 was set as the cutoff criterion.

### Construction of risk assessment model and risk score calculation

To further screen for prognosis-related methylation-driven genes, a linear risk assessment model for LUAD methylation-driven genes was constructed using a single-factor, multivariate Cox analysis [25]. The prognostic index was defined as follows:

$$ \text{Prognostic index} = \sum\limits_{i = 1}^{N} {Exp_{i} \times \beta_{i}}. $$

In the formula, Exp is the expression level of each methylation-driven gene in the specimen, and β is the multi-factor COX regression analysis coefficient of each methylation-driven gene in the COX model. The prognostic risk value of each sample was calculated according to the formula, and then the median of the index value was cut off. The patients were divided into high and low risk groups [26, 27] and verified with a time-dependent ROC curve. The Kaplan–Meier survival curve method was used to evaluate the overall survival rate of patients in the high- and low-risk groups. Log-rank test was used to determine whether there was any difference in the overall survival rate between the high-risk and low-risk groups. P < 0.05 was considered statistically significant. In addition, we applied the risk prediction model to patients with stage I LUAD who had both the expression and clinical information provided by the TCGA database to further test the validity and practicability of the model to predict prognosis.

### Mapping of Kaplan–Meier curves of driver genes and methylated sites in survival models, joint survival analysis

To further explore the prognostic assessment of methylation-driven genes, we extracted relevant loci for driving gene methylation from downloaded LUAD methylation data. Then, based on the survival R package, the prognostic survival analysis of the driving genes and related methylated sites was performed by combining the clinical data and prognostic information of LUAD in TCGA, and the Kaplan–Meier curve was produced. In addition, we performed a joint survival analysis of methylation levels and gene expression levels of driver genes to further identify key genes associated with prognosis in patients with LUAD, and the joint survival curve was also obtained by the survival R package.

## Results

### TCGA data analysis and acquisition of methylation-driven genes

**Fig. 1:**
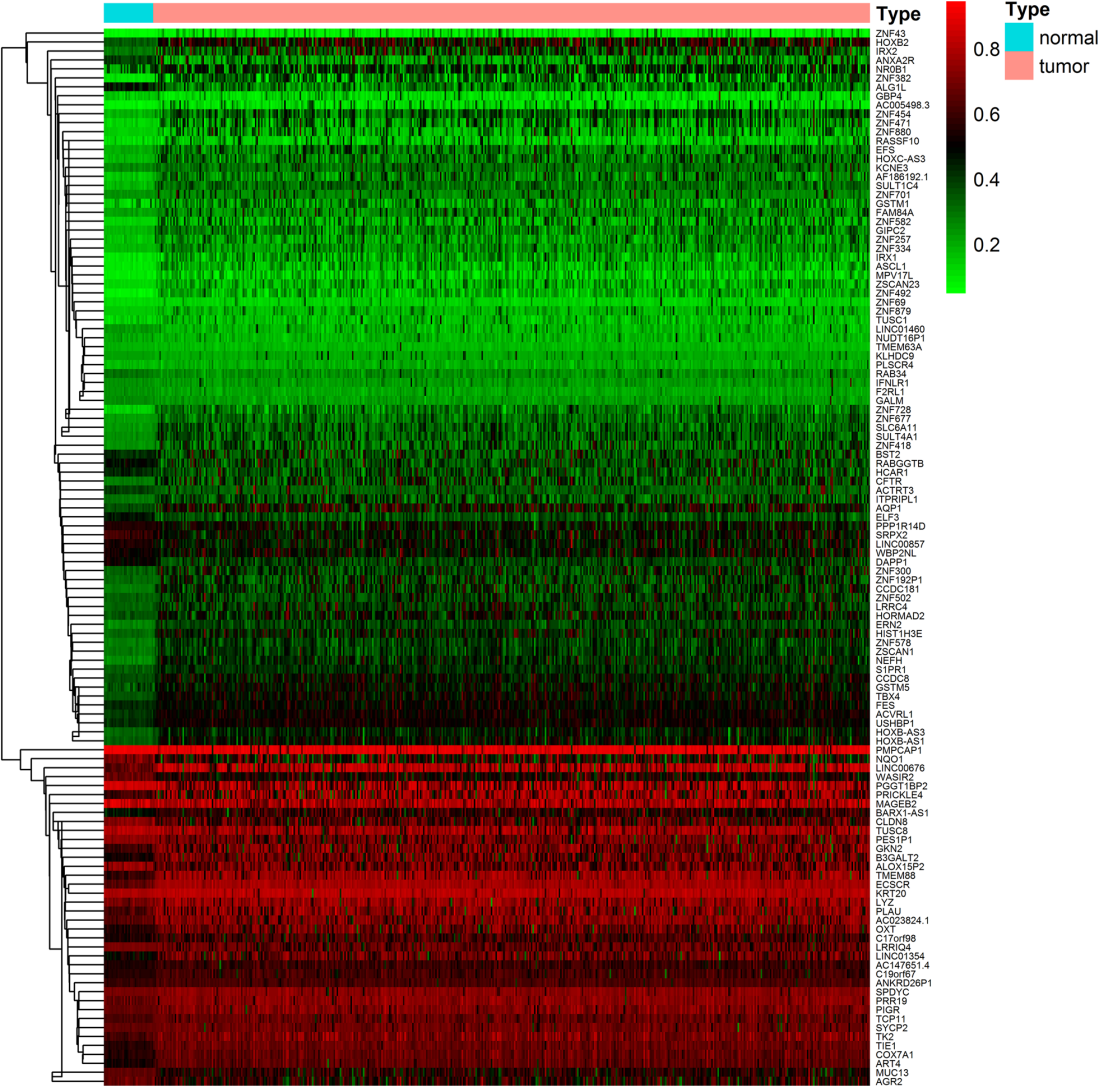
Heat maps of LUAD-related aberrant methylation-driven genes. The color from green to red shows a trend from hypomethylation to hypermethylation

The methylation data from the analysis of this study were from 507 sample data, including 475 LUAD samples and 32 paracancerous control samples. Gene expression data were gathered from 594 samples, including 535 cancer samples and 59 paracancerous control samples. Abnormal methylation expression and gene expression data of LUAD from the TCGA were extracted and analyzed based on the LIMMA software package. The relevant data were then integrated under the same sample based on the MethylMix package for correlation analysis. A mixed model construction and Wilcoxon rank test for differential methylation were calculated, where |logFC| > 0, P < 0.05, |Cor| > 0.3. As a screening condition, 118 methylation-driven genes were obtained (Fig. 1; Additional files 1, 2, 3).

### Functional enrichment and pathway analyses of methylation-driven genes

**Fig. 2:**
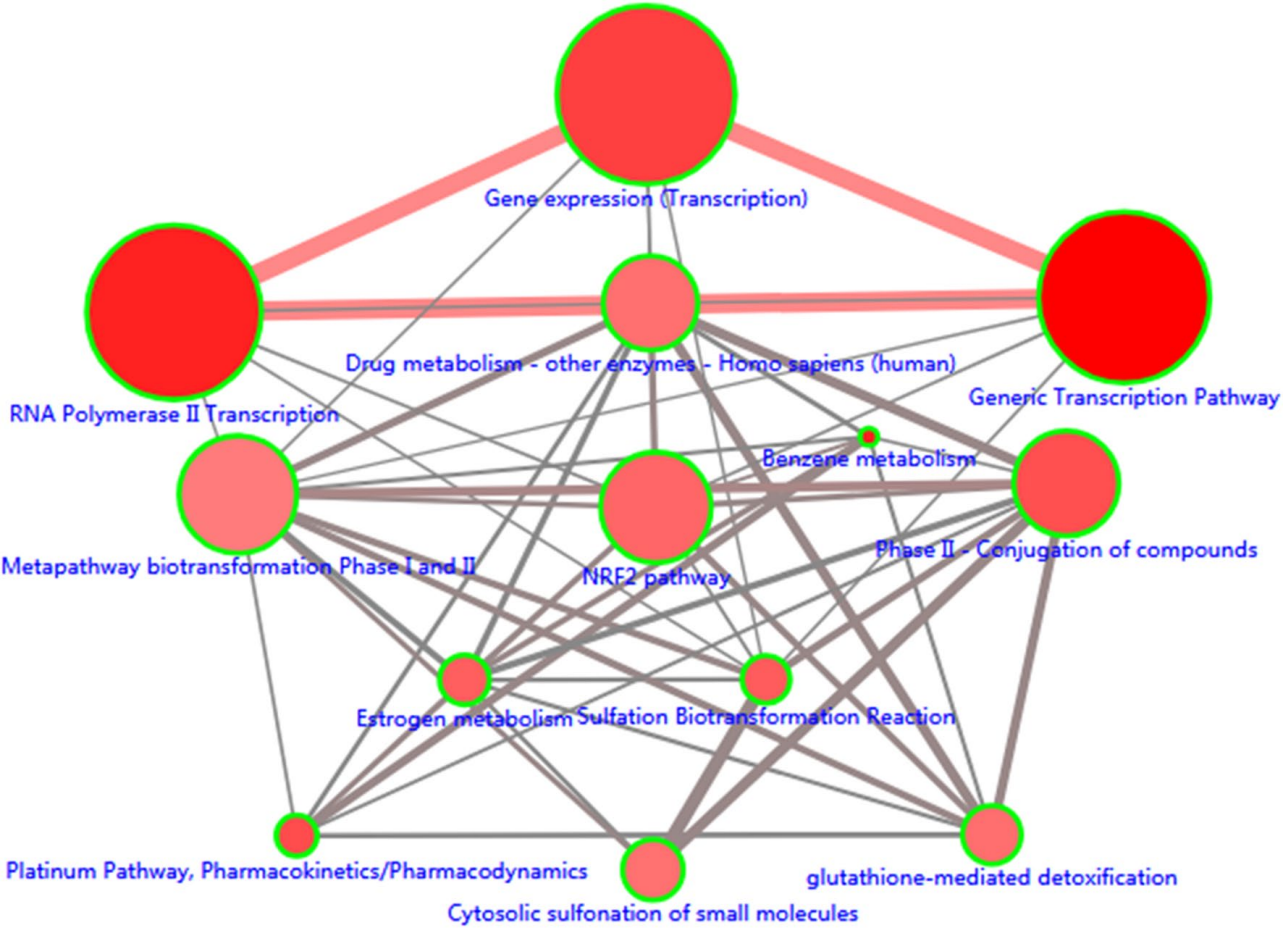
The significant enriched pathways of methylation-driven genes. Only the pathways which P < 0.01 were shown here. Node size: the number of genes; Node color: P-value; Edge width: percentage of shared genes; Edge color: genes from input

To further explore the molecular mechanism of methylation-driven genes in the development of LUAD, we performed a functional enrichment analysis and pathway analysis of these genes using DAVID and ConsensusPathDB online software. The results showed that the methylation-driven genes were enriched not only in multiple pathways but also in molecular functions and biological processes. Pathway analysis showed that these genes were mainly enriched in the generic transcription, RNA polymerase II transcription, gene expression (transcription) and platinum pathways, as well as pharmacokinetics/pharmacodynamics (Fig. 2; Additional file 4). Functional analysis revealed that, in the biological processes (BP) group, these genes were mainly involved in transcriptional regulation, DNA-templates, multicellular organism development, angiogenesis and blood vessel maturation, among others. The molecular function (MF) was mainly enriched in transcription factor activity, sequence-specific DNA binding, nucleic acid binding, and DNA binding. In addition, the cellular component (CC) group was mainly involved in tracellularly (Table 1).

**Table 1:** Functional enrichment analysis of methylation-driven genes associated with LUAD

Category	Term	Count	P value
GOTERM_BP_DIRECT	GO:0006355~regulation of transcription, DNA-templated	26	1.49E−08
	GO:0006351~transcription, DNA-templated	23	8.28E−05
	GO:0001525~angiogenesis	6	0.004235
	GO:0072272~proximal/distal pattern formation involved in metanephric nephron development	2	0.009506
	GO:0032526~response to retinoic acid	3	0.016299
GOTERM_MF_DIRECT	GO:0003700~transcription factor activity, sequence-specific DNA binding	17	1.77E−05
	GO:0003676~nucleic acid binding	16	9.29E−05
	GO:0046872~metal ion binding	21	0.002409
	GO:0003677~DNA binding	17	0.007796
GOTERM_CC_DIRECT	GO:0005622~intracellular	16	0.001325

If there were more than five terms in this category, selected the first five terms based on the P value. Count: the number of enriched genes in each term

### Construction and analysis of the prognosis risk assessment model of LUAD methylation-driven genes

**Fig. 3:**
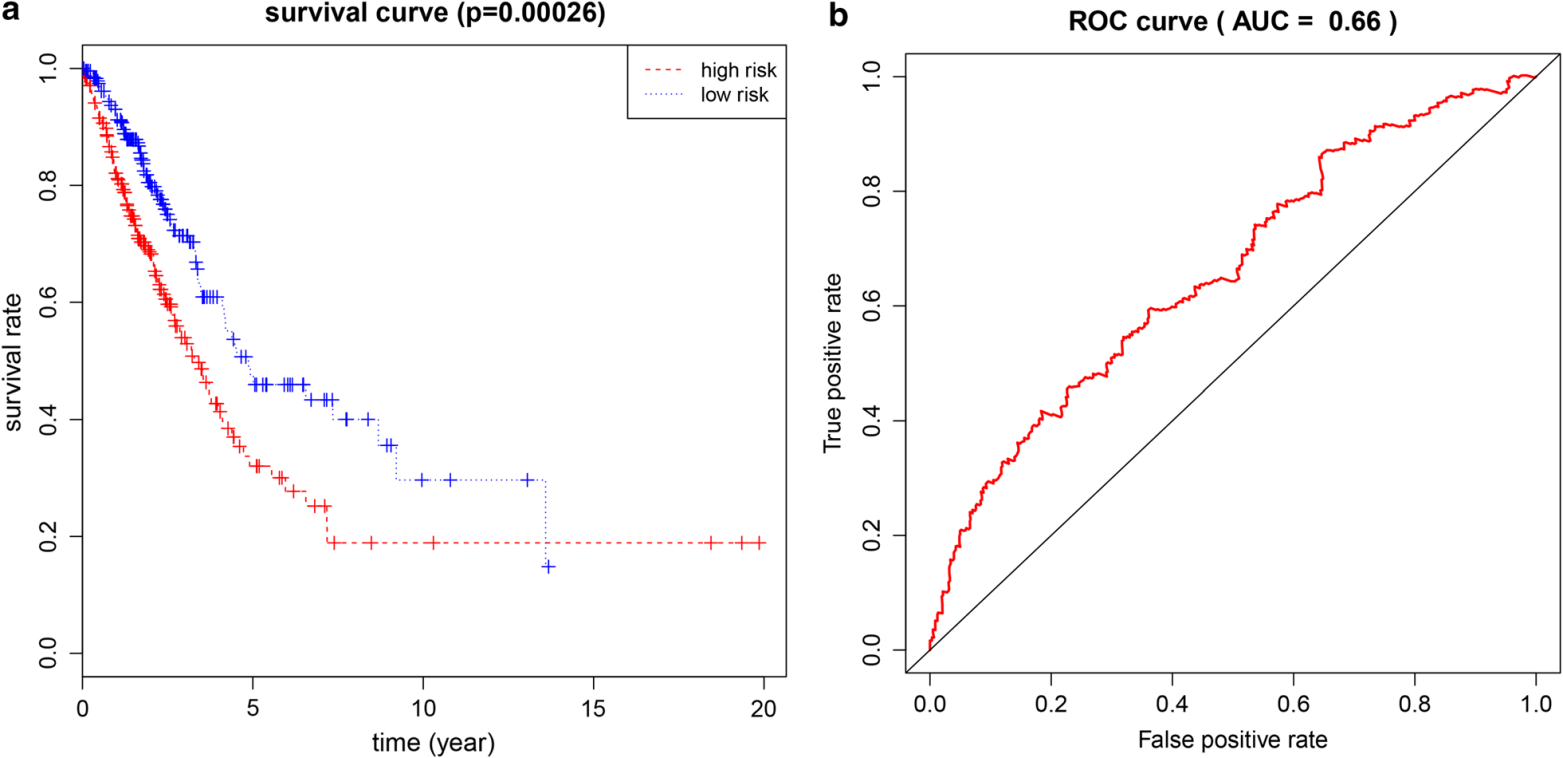
Kaplan–Meier and ROC curves for a linear risk model based on five methylation-driven genes. **a** The differences between the high-risk (n = 244) and low-risk (n = 244) groups were determined by the log-rank test. **b** Time-dependent ROC curves analysis for 3-year survival prediction by methylation-driven genes

**Fig. 4:**
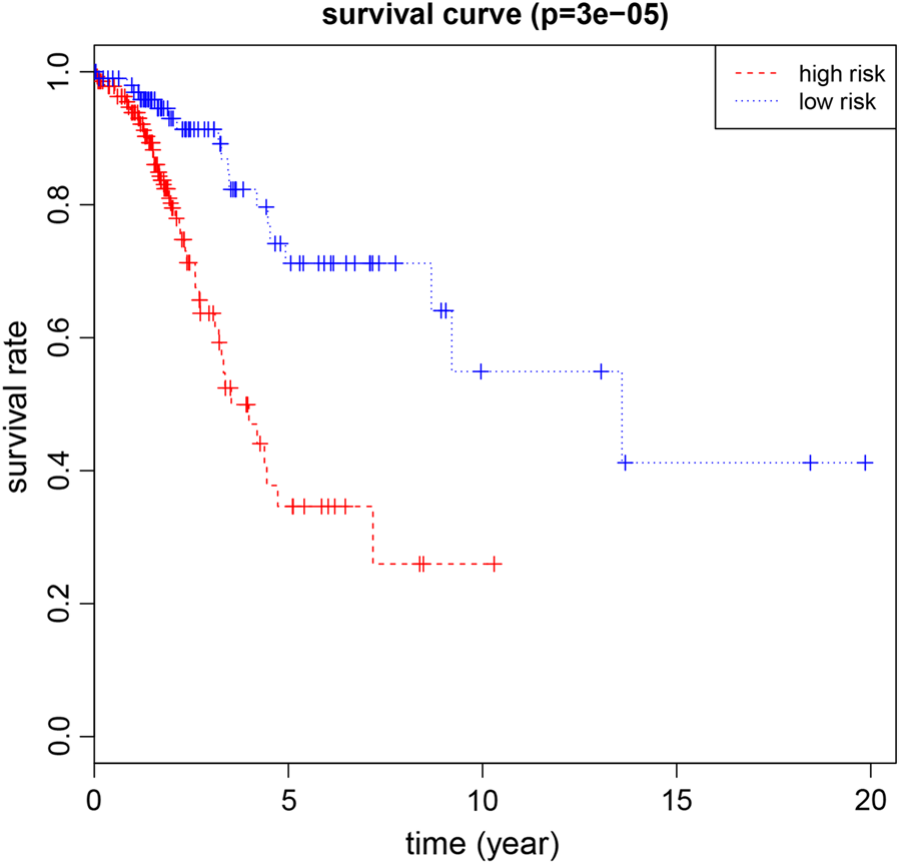
Kaplan–Meier curve for patients with stage I LUAD using the prognostic risk model

We performed univariate and multivariate Cox regression analyses of the obtained LUAD methylation-driven genes. The results showed that the assessment model constructed by five genes (CCDC181, PLAU, S1PR1, ELF3, KLHDC9) can be used as an independent indicator to predict the prognosis of the disease. The prognostic index = (1.571 * expression level of CCDC181) + (− 1.170 * expression level of PLAU) + (3.674 * expression level of S1PR1) + (3.467 * expression level of ELF3) + (3.028 * expression level of KLHDC9). In addition, with a median PI value (value = 0.928) as a group cutoff condition, 488 samples in the methylated data sample that matched the clinical follow-up sample were divided into a high-risk group (n = 244) and a low-risk group (n = 244). Kaplan–Meier survival curve analysis of patients in the high- and low-risk groups showed that the overall survival rate was lower in the high-risk group, and the difference between the two groups was statistically significant (Fig. 3a). Using the time-dependent ROC curve to estimate the predictive performance of the risk scoring model, the AUC of the prognostic risk assessment model for the five methylation-driven genes was 0.66 at 3 years of OS (Fig. 3b). In the prognostic assessment test for patients with stage I LUAD, the Kaplan–Meier curve showed that the survival rate of the high-risk group was significantly lower than that of the low-risk group (Fig. 4). It can be seen that the model based on methylation-driven genes has certain reliability and practicability in evaluating the prognosis of LUAD patients.

### Survival analysis of driving gene methylated sites and joint survival analysis of methylation and expression

**Fig. 5:**
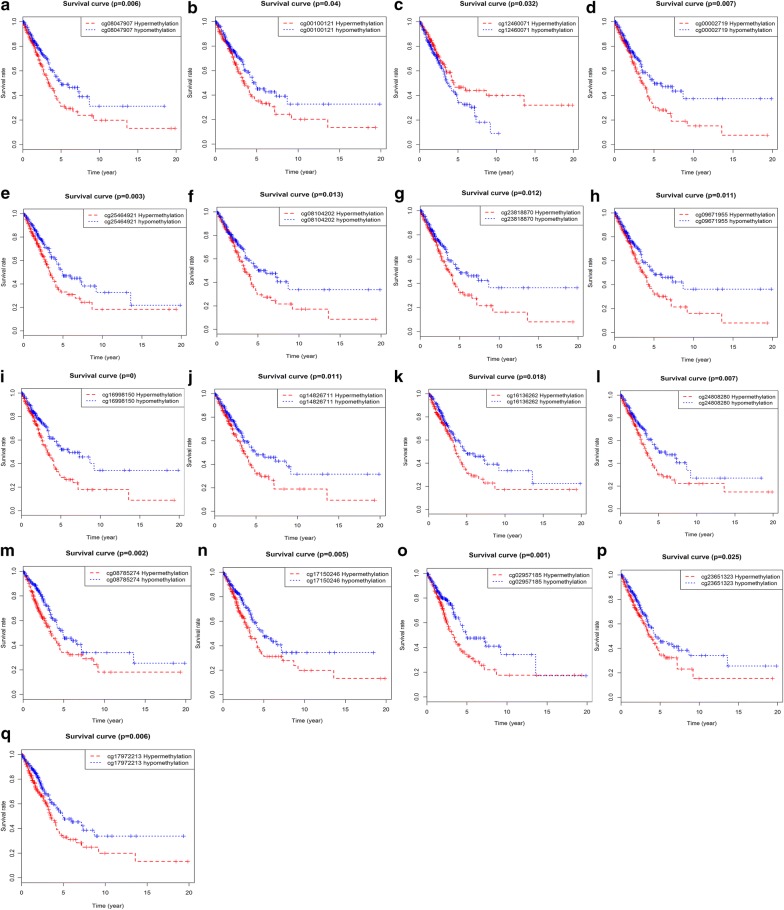
Kaplan–Meier survival curves of the related methylated sites. **a**–**l** Methylated sites of the gene CCDC181; **m**–**p** methylated sites of the gene KLHDC9; **q** methylated site of the gene S1PR1

**Fig. 6:**
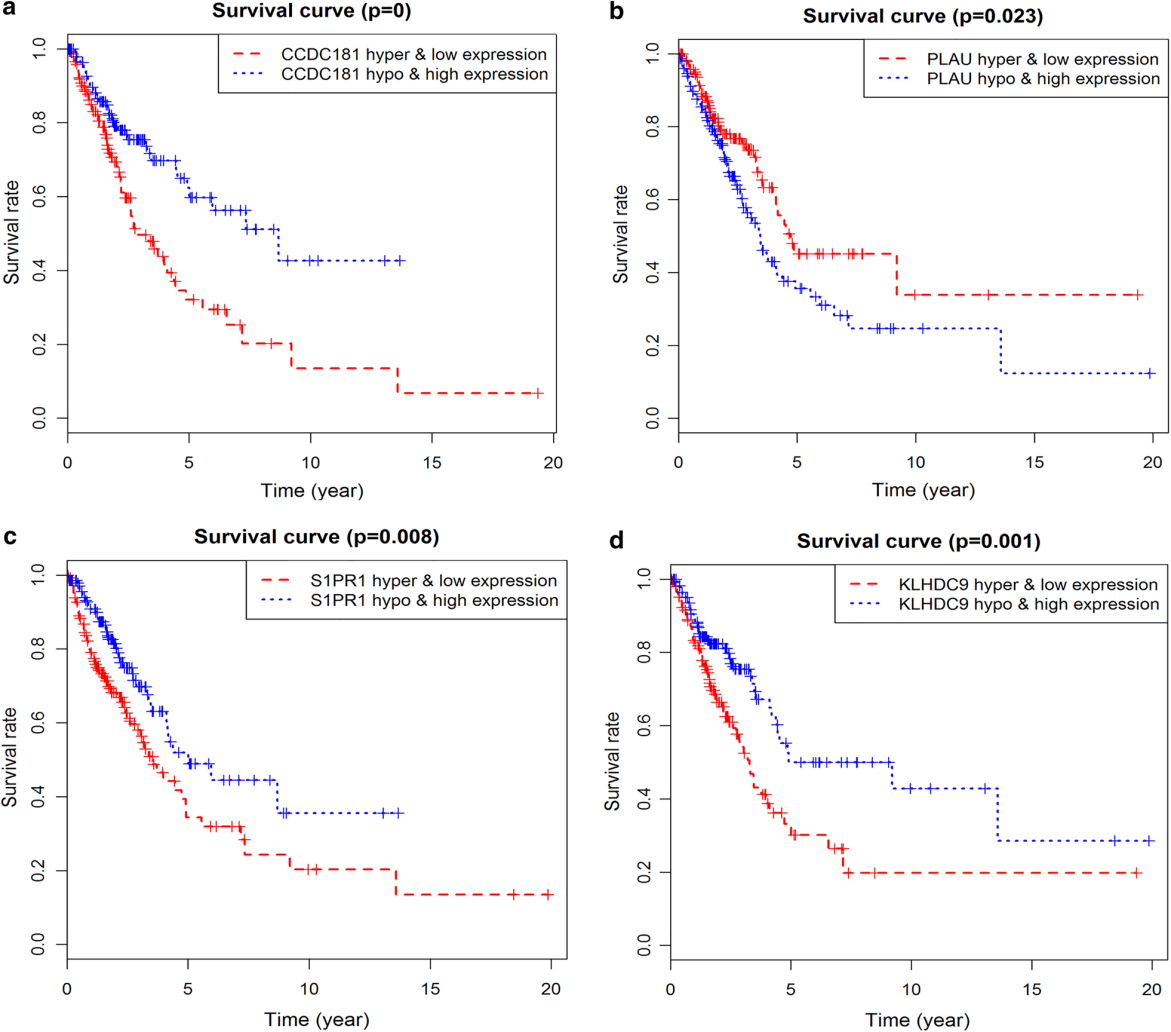
Kaplan–Meier survival curves for the joint survival analysis. **a** The combination of gene CCDC181 methylation and expression; **b** the combination of gene PLAU methylation and expression; **c** the combination of gene S1PR1 methylation and expression; **d** the combination of gene KLHDC9 methylation and expression

Prognostic survival analysis of driver gene-related methylated sites in a risk assessment model was performed based on the surviving R package. P < 0.05 was used as a meaningful cut-off condition for predicting prognosis, and we found specific methylated sites associated with the prognosis of these genes (Additional file 5). Among them, 12 methylated sites of the gene CCDC181, 1 methylated site of S1PR1, and 4 methylated sites of KLHDC9 were significantly associated with the prognosis of LUAD (Fig. 5). In addition, joint survival analysis revealed that the combination of methylation and expression of the genes CCDC181, S1PR1, KLHDC9, and PLAU had a significant correlation with the prognosis of the patient, including the gene CCDC181. The high-methylation low-expression survival rate of S1PR1 and KLHDC9 were higher, while the low-methylation high-expression survival rate of the gene PLAU was higher (Fig. 6).

## Discussion

LUAD is the most common type of lung cancer, and it is highly invasive and has a poor prognosis [28, 29]. In-depth studies on the molecular pathogenesis of LUAD and the early detection of the prognostic markers of this disease as well as specific driving genes are urgent and could prove useful for improving patients’ quality of life and the prognosis of patients. In recent years, it has been found that the decreased expression of genes caused by the hypermethylation and the enhanced expression of genes caused by hypomethylation play an important role in the regulation and development of malignant tumors [9, 30]. Gene methylation could lead to transcriptional disorders, causing some gene expression disorders and cell differentiation disorders [11, 31]. Studies have shown that, unlike DNA aberrations, epigenetic changes are reversible, making them attractive therapeutic targets [32]; thus, detecting and altering DNA methylation can provide new insights for the further treatment and risk assessment of cancer. For instance, Robles et al. found that methylation of the HOXA9 promoter was associated with a high risk in patients with stage I LUAD [33], whereas Sugimoto et al. found that the aberrant methylation of GRWD1 may be a protective factor in tumor development. Because the high expression of GRWD1 in tumor cells can promote tumor cell growth, the aberrant methylation of the GRWD1 gene can inhibit its activity [34]. From previous studies, the prognosis-related methylation genes and specific methylation-driven genes could serve as the new markers for further clinical applications.

In the present study, our aim was to screen for methylation-driven genes by analyzing data on LUAD methylation in TCGA and to identify new prognostic biomarkers associated with methylation. Firstly, we obtained 118 methylation-driven genes using the LIMMA software package. And then, to further investigate the cellular mechanisms involved in these methylation-driven genes, functional enrichment and pathway analyses were performed. Functional aggregation analysis revealed that these genes are involved intracellularly and that major enrichment is associated with the regulation of transcription, DNA-templates, multicellular organism development, and a variety of other functions, such as transcription factor activity and sequence-specific DNA binding. Enrichment pathway analyses indicated that the methylation-driven genes were mainly involved in the generic transcription, RNA polymerase II transcription, gene expression (transcription) and platinum pathways. These functional enrichment and pathway analyses demonstrate not only the cellular mechanisms by which DNA methylation abnormalities lead to disease development but also the functional level interaction of these genes.

In addition, according to univariate and multivariate Cox analyses of methylation-driven genes, five methylation-driven genes (CCDC181, PLAU, S1PR1, ELF3, KLHDC9) were identified and used to construct a prognostic risk model for LUAD. Using this risk model, we successfully divided the LUAD samples into high-risk and low-risk groups. Survival analysis showed significant differences in overall survival between the two groups. The results suggest that the risk model, consisting of five methylation-driven gene profiles, can effectively predict the prognosis of patients with LUAD. The results also show that the AUC of the ROC curve predicting 3-year survival by the five-gene feature was 0.66. Our study found that the risk assessment models constructed by these five gene signatures performed well in survival predictions for patients with LUAD, but further studies are needed to validate these findings. These methylation-driven genes can be used as effective biomarkers or drug targets for early diagnosis, and prognosis of patients with LUAD. C1orf114, also known as CCDC181, is a protein of unknown function and encodes a coiled-coil domain containing 181. Haldrup et al. showed that CCDC181 (C1orf114) and HAPLN3 can be used as novel biomarkers for the hypermethylation of prostate cancer (PC), proving to be a novel diagnostic and/or prognostic promoter. Moreover, the combination of C1orf114 and two other genes in the dichotomy methylation profile (AOX1 and HAPLN3) can increase the prognostic value of PC [35]. Our study found that CCDC181 is one of the methylation-driven genes of LUAD, and its hypermethylation leads to poor prognosis. The molecular mechanism of the gene CCDC18 in cancer requires further research to discover its driving genetic role in LUAD patients.

The gene PLAU is a urokinase plasminogen activator. Based on the established role of the plasminogen system in cancer invasiveness and as a key player in cancer metastasis and cancer cell invasion behavior (adhesion, migration and invasion) [36, 37], PLAU has different expression levels in individual cells of lung cancer patients and can be used as a marker to predict the prognosis of lung cancer patients [38]. Methylation and gene expression combined survival analyses found that the gene PLAU were closely associated with poor prognosis in patients with LUAD. Sphingosine 1-phosphate receptor 1 (S1PR1) is a G protein-coupled receptor involved in the regulation of physiological processes such as cell growth, differentiation, and migration as well as the immune response [39]. Previous studies have shown that S1PR1 can participate in the proliferation and invasion of cancer cells by activating the ERK signaling pathway [40]. Moreover, S1PR1 can stably activate STAT3 in tumor and bone marrow cells [41, 42], which is essential for breast cancer cell proliferation, invasion and metastasis. In addition, Yoshida et al. showed that S1PR1 expression was associated with prognosis in patients with glioblastoma and that high expression was positively correlated with favorable survival [43]. This study found that the high methylation level of S1PR1 was associated with a lower survival rate in patients with LUAD and may be a new target for the treatment and improvement of the prognosis of patients with LUAD.

In addition, as a potential oncogenic transcription factor, ELF3 promotes tumor cell growth and metastasis by modulating the PI3K/Akt and ERK pathways in NSCLC and may serve as a promising new target for the treatment of NSCLC patients [44, 45]. Other studies have shown that ELF3 overexpression is significantly associated with poor prognosis in patients with hepatocellular carcinoma (HCC) [46]. In our study, the gene KLHDC9 was used as one of the prognostic-related methylation-driven genes in LUAD, and the KM curve showed that KLHDC9 hypermethylation patients had shorter survival times and shorter methylation survival times. The combined survival analysis also found that hypermethylation and low gene expression of KLHDC9 suggested poor prognosis in patients with LUAD. There are few studies that have examined the gene KLHDC9, but this gene could be used as a new target for the diagnosis and prognosis of LUAD. However, based on the fact that DNA methylation mainly occurs at the CpG site of the gene, this study found that multiple CpG sites were closely related to the prognosis of patients with LUAD by analyzing the correlation of CpG site survival in methylation-driven genes. These results are based on bioinformatic analysis and provide a theoretical basis for further experimental validation.

## Conclusion

In this study, based on the genomic methylation data provided by TCGA for LUAD patients, we obtained 118 methylation-driven genes associated with LUAD using the MethylMix algorithm. Univariate and multivariate Cox regression analyses showed that the prognostic survival model constructed from five aberrant methylation-driven genes, CCDC181, PLAU, S1PR1, ELF3, and KLHDC9, was an independent predictor of disease prognosis including. Based on the risk model of these five methylation-driven genes, LUAD patients can be divided into a high-risk group and low-risk group, and this approach provides a basis for prognosis prediction and personalized treatment plans for LUAD patients. And the predictive ability of the model was further demonstrated in the test of stage I LUAD samples. Since DNA methylation is one of the most important modifications in epigenetics, gene expression can be controlled in a variety of ways. Joint survival analysis revealed that the expression levels of the genes CCDC181, PLAU, S1PR1 and KLHDC9 can be used as independent prognostic markers or drug targets for early diagnosis and prognosis assessment of LUAD. Although further experimental verification is needed, our findings provide an important bioinformatic basis and relevant theoretical basis for guiding the subsequent in-depth study of LUAD.

## Additional files

**Additional file 1.** Methylation levels of 118 driver genes.

**Additional file 2.** Expression levels of 118 methylation-driven genes.

**Additional file 3.** The protein–protein interaction network of 118 methylation-driven genes and the related genes. Red: the methylation-driven genes; Green: the related genes. The methylation-driven genes which not associated with other genes are not shown here.

**Additional file 4.** Pathway analysis of methylation-driven genes associated with LUAD (P < 0.05).

**Additional file 5.** All relevant methylated sites of the five methylation-driven genes obtained from the TCGA database. (1–16) methylated sites of the gene CCDC181; (17–33) methylated sites of the gene ELF3; (34–47) methylated sites of the gene KLHDC9; (48) methylated site of the gene PLAU; (49–57) methylated sites of the gene S1PR1.

## Abbreviations

LUAD: lung adenocarcinoma
NSCLC: non-small cell lung cancer
TCGA: The Cancer Genome Atlas
BP: biological processes
MF: molecular function
CC: cellular component
S1PR1: Sphingosine 1-phosphate receptor 1

## References

[1] Jemal A, Bray F, Center M, Ferlay J, Ward E, Forman D (2011). Global cancer statistics. CA Cancer J Clin.

[2] Dias M, Linhas R, Campainha S, Conde S, Barroso A (2017). Lung cancer in never-smokers—what are the differences?. Acta Oncol.

[3] Sun S, Schiller J, Gazdar A (2007). Lung cancer in never smokers—a different disease. Nat Rev Cancer.

[4] Yoshizawa A, Motoi N, Riely G, Sima C, Gerald W, Kris M, Park B, Rusch V, Travis W (2011). Impact of proposed IASLC/ATS/ERS classification of lung adenocarcinoma: prognostic subgroups and implications for further revision of staging based on analysis of 514 stage I cases. Mod Pathol.

[5] Sakashita S, Sakashita M, Sound Tsao M (2014). Genes and pathology of non-small cell lung carcinoma. Semin Oncol.

[6] Raso M, Behrens C, Herynk M, Liu S, Prudkin L, Ozburn N, Woods D, Tang X, Mehran R, Moran C (2009). Immunohistochemical expression of estrogen and progesterone receptors identifies a subset of NSCLCs and correlates with EGFR mutation. Clin Cancer Res.

[7] Siegelin M, Borczuk A (2014). Epidermal growth factor receptor mutations in lung adenocarcinoma. Lab Invest.

[8] Song J, Ye A, Jiang E, Yin X, Chen Z, Bai G, Zhou Y, Liu J (2018). Reconstruction and analysis of the aberrant lncRNA–miRNA–mRNA network based on competitive endogenous RNA in CESC. J Cell Biochem.

[9] Meissner A, Lander E (2007). The mammalian epigenome. Cell.

[10] Beland F (2009). DNA hypomethylation in the origin and pathogenesis of human diseases. Cell Mol Life Sci.

[11] Laurent L, Ren B, Loring J, Fan J (2008). Unraveling epigenetic regulation in embryonic stem cells. Cell Stem Cell.

[12] Kulis M, Esteller M (2010). DNA methylation and cancer. Adv Genet.

[13] Feng H, Zhang Z, Wang X, Liu D (2016). Identification of DLC-1 expression and methylation status in patients with non-small-cell lung cancer. Mol Clin Oncol.

[14] Guo F, Guo L, Li Y, Zhou Q, Li Z (2015). MALAT1 is an oncogenic long non-coding RNA associated with tumor invasion in non-small cell lung cancer regulated by DNA methylation. Int J Clin Exp Pathol.

[15] Li J, Jia X, Liu J, Liu J, Zhao H (2015). Relationship of EGFR DNA methylation with the severity of non-small cell lung cancer. Genet Mol Res.

[16] Saito K, Kawakami K, Matsumoto I, Oda M, Watanabe G, Minamoto T (2010). Long interspersed nuclear element 1 hypomethylation is a marker of poor prognosis in stage IA non-small cell lung cancer. Clin Cancer Res.

[17] Feng H, Zhang Z, Qing X, Wang X, Liang C, Liu D (2016). Promoter methylation of APC and RAR-β genes as prognostic markers in non-small cell lung cancer (NSCLC). Exp Mol Pathol.

[18] Zhang X, Yang X, Wang J, Liang T, Gu Y, Yang D (2015). Down-regulation of PAX6 by promoter methylation is associated with poor prognosis in non small cell lung cancer. Int J Clin Exp Pathol.

[19] Tomczak K, Czerwińska P, Wiznerowicz M (2015). The Cancer Genome Atlas (TCGA): an immeasurable source of knowledge. Contemp Oncol (Pozn).

[20] Gevaert O (2015). MethylMix: an R package for identifying DNA methylation-driven genes. Bioinformatics.

[21] Gevaert O, Tibshirani R, Plevritis S (2015). Pancancer analysis of DNA methylation-driven genes using MethylMix. Genome Biol.

[22] Huang DW, Sherman B, Lempicki R (2009). Systematic and integrative analysis of large gene lists using DAVID bioinformatics resources. Nat Protoc.

[23] Kamburov A, Pentchev K, Galicka H, Wierling C, Lehrach H, Herwig R (2011). ConsensusPathDB: toward a more complete picture of cell biology. Nucleic Acids Res.

[24] Kamburov A, Stelzl U, Lehrach H, Herwig R (2013). The ConsensusPathDB interaction database: 2013 update. Nucleic Acids Res.

[25] Lossos I, Czerwinski D, Alizadeh A, Wechser M, Tibshirani R, Botstein D, Levy R (2004). Prediction of survival in diffuse large-B-cell lymphoma based on the expression of six genes. N Engl J Med.

[26] Bao Z, Li M, Wang J, Zhang C, Wang H, Yan W, Liu Y, Zhang W, Chen L, Jiang T (2014). Prognostic value of a nine-gene signature in glioma patients based on mRNA expression profiling. CNS Neurosci Ther.

[27] Cheng W, Ren X, Cai J, Zhang C, Li M, Wang K, Liu Y, Han S, Wu A (2015). A five-miRNA signature with prognostic and predictive value for MGMT promoter-methylated glioblastoma patients. Oncotarget.

[28] Gardiner N, Jogai S, Wallis A (2014). The revised lung adenocarcinoma classification—an imaging guide. J Thorac Dis.

[29] Sardenberg R, Mello E, Younes R (2014). The lung adenocarcinoma guidelines: what to be considered by surgeons. J Thorac Dis.

[30] Dobersch S, Romero-Olmedo A, Barreto G (2015). Epigenetics in lung cancer diagnosis and therapy. Cancer Metast Rev.

[31] Chang S, Wang H, Yang S, Lai K, Lee T (2018). Prognostic values of EPDR1 hypermethylation and its inhibitory function on tumor invasion in colorectal cancer. Cancers.

[32] Baylin S, Jones P (2011). A decade of exploring the cancer epigenome—biological and translational implications. Nat Rev Cancer.

[33] Robles A, Arai E, Mathé E, Okayama H, Schetter A, Brown D, Petersen D, Bowman E, Noro R, Welsh J (2015). An integrated prognostic classifier for stage I lung adenocarcinoma based on mRNA, microRNA, and DNA methylation biomarkers. J Thorac Oncol.

[34] Sugimoto N, Maehara K, Yoshida K, Yasukouchi S, Osano S, Watanabe S, Aizawa M, Yugawa T, Kiyono T, Kurumizaka H (2015). Cdt1-binding protein GRWD1 is a novel histone-binding protein that facilitates MCM loading through its influence on chromatin architecture. Nucleic Acids Res.

[35] Haldrup C, Mundbjerg K, Vestergaard E, Lamy P, Wild P, Schulz W, Arsov C, Visakorpi T, Borre M, Høyer S (2013). DNA methylation signatures for prediction of biochemical recurrence after radical prostatectomy of clinically localized prostate cancer. J Clin Oncol.

[36] Han B, Nakamura M, Mori I, Nakamura Y, Kakudo K (2005). Urokinase-type plasminogen activator system and breast cancer (Review). Oncol Rep.

[37] Sliva D (2008). Suppression of cancer invasiveness by dietary compounds. Mini Rev Med Chem.

[38] Di Bernardo M, Matakidou A, Eisen T, Houlston R (2009). Plasminogen activator inhibitor variants PAI-1 A15T and PAI-2 S413C influence lung cancer prognosis. Lung Cancer.

[39] Mendelson K, Evans T, Hla T (2014). Sphingosine 1-phosphate signalling. Development.

[40] Li M, Sanchez T, Yamase H, Hla T, Oo M, Pappalardo A, Lynch K, Lin C, Ferrer F (2009). S1P/S1P1 signaling stimulates cell migration and invasion in Wilms tumor. Cancer Lett.

[41] Degagné E, Pandurangan A, Bandhuvula P, Kumar A, Eltanawy A, Zhang M, Yoshinaga Y, Nefedov M, de Jong P, Fong L (2014). Sphingosine-1-phosphate lyase downregulation promotes colon carcinogenesis through STAT3-activated microRNAs. J Clin Invest.

[42] Deng J, Liu Y, Lee H, Herrmann A, Zhang W, Zhang C, Shen S, Priceman S, Kujawski M, Pal S (2012). S1PR1-STAT3 signaling is crucial for myeloid cell colonization at future metastatic sites. Cancer Cell.

[43] Yoshida Y, Nakada M, Harada T, Tanaka S, Furuta T, Hayashi Y, Kita D, Uchiyama N, Hayashi Y, Hamada J (2010). The expression level of sphingosine-1-phosphate receptor type 1 is related to MIB-1 labeling index and predicts survival of glioblastoma patients. J Neurooncol.

[44] Wang H, Yu Z, Huo S, Chen Z, Ou Z, Mai J, Ding S, Zhang J (2018). Overexpression of ELF3 facilitates cell growth and metastasis through PI3K/Akt and ERK signaling pathways in non-small cell lung cancer. Int J Biochem Cell Biol.

[45] Zhang D, Qu L, Ma L, Zhou Y, Wang G, Zhao X, Zhang C, Zhang Y, Wang M, Zhang M (2018). Genome-wide identification of transcription factors that are critical to non-small cell lung cancer. Cancer Lett.

[46] Zheng L, Xu M, Xu J, Wu K, Fang Q, Liang Y, Zhou S, Cen D, Ji L, Han W (2018). ELF3 promotes epithelial–mesenchymal transition by protecting ZEB1 from miR-141-3p-mediated silencing in hepatocellular carcinoma. Cell Death Dis.

