# Supplementary figures and images for: Exploration of methylation-driven genes for monitoring and prognosis of patients with lung adenocarcinoma
Source: Cancer Cell Int. 2018 Nov 26;18:194. doi: 10.1186/s12935-018-0691-z (PMC6258452; doi:10.1186/s12935-018-0691-z)

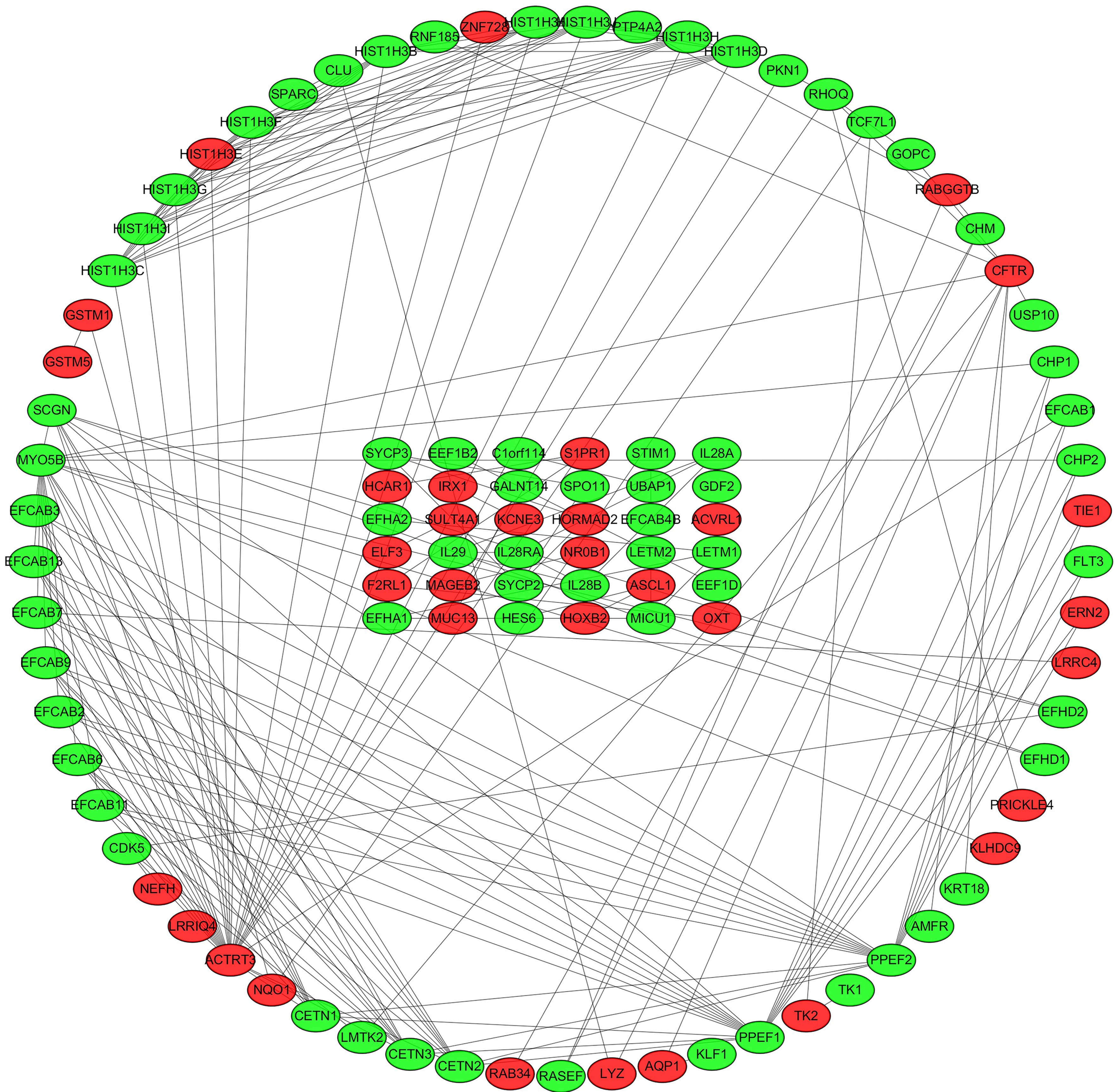

Supplement: Supplementary file 3 — Additional file 3. The protein–protein interaction network of 118 methylation-driven genes and the related genes. Red: the methylation-driven genes; Green: the related genes. The methylation-driven genes which not associated with other genes are not shown here. [file 12935_2018_691_MOESM3_ESM.pdf]
